# A Case Report of Opisthotonos Associated with Administration of Intramuscular Ketamine

**DOI:** 10.5811/cpcem.2021.6.53069

**Published:** 2021-10-19

**Authors:** Matthew W. Morgan, Ellen Dore

**Affiliations:** Regions Hospital, Department of Emergency Medicine, St. Paul, Minnesota

**Keywords:** case report, ketamine, opisthotonos

## Abstract

**Introduction:**

Ketamine, a commonly used medication to treat agitation, has known adverse effects such as emergence reactions, vomiting, and laryngospasm. Opisthotonos has not been a commonly reported adverse reaction.

**Case Report:**

We report a case of opisthotonos brought on by administration of ketamine. A 24-year-old male with a history of schizophrenia was brought in by emergency medical services with opisthotonos shortly after treatment with 250 milligrams intramuscular ketamine by paramedics. He had become increasingly paranoid after being off his aripiprazole for a few weeks, and his family had become afraid for his and their safety. Paramedics administered ketamine to control his combative agitation, per protocol. The patient’s extreme neck and back extension rapidly resolved with the administration of midazolam. Further history and workup did not reveal another cause for opisthotonos.

**Conclusion:**

This is the first reported case to our knowledge of ketamine-associated opisthotonos in the emergency setting. Emergency care providers should be aware of this potential side effect.

## INTRODUCTION

Ketamine has been shown to be a safe and effective medication in the prehospital setting for patients requiring emergent sedation.^1^ Common side effects include emergence reactions, post-sedation nausea, hypertension, and tachycardia. We present a unique case of opisthotonos that occurred after intramuscular ketamine administration.

## CASE REPORT

A 24-year-old man was brought in by paramedics in apparent extremis after having received a 250-milligram (mg) intramuscular injection of ketamine. Paramedics had been called by family because the patient, who had a history of schizophrenia, had become increasingly paranoid and religiously preoccupied, and his family had become afraid for his and their safety. The family later provided the history that he had been eating and sleeping minimally. That day he had been shaking uncontrollably on the floor while talking about being possessed. He thought that his medications were poisoning him and thus had been off them for three to four weeks. The paramedics were unable to transport the patient safely and had given him the ketamine to control his agitation per their protocol.

On arrival to the emergency department (ED) the patient displayed opisthotonos, his back consistently held arched in extension. He spoke no discernible words but rather mumbled gibberish or moaned. His eyes were closed initially. When opened by the provider, rotary nystagmus was noted, with normal size and sluggish pupils. He showed no meaningful response to verbal stimuli. His initial vital signs showed him to be tachycardic at 140 beats per minute, hypertensive at 153/100 millimeters mercury, and tachypneic at 30 breaths per minute, but afebrile, with normal oxygen saturations in the upper nineties on room air. His exam did not reveal signs of trauma. His lungs were clear, and he had no rigidity or clonus. He would occasionally flex his upper extremities such as with stimuli from intravenous line placement, but maintained extended, arched posture.

The patient was immediately given 2.5 mg of midazolam intravenously, which partially relieved his opisthotonos within a few minutes. Instead of nearly constant extensor posturing of the neck it became more intermittent, and arching of the back lessened. A few minutes later he was given an additional 2.5 mg of midazolam, and a few minutes thereafter, cessation of the opisthotonos was noted. His heart rate declined to between 100–110 beats per minute. His breathing also notably slowed to a rate between 20–25 respirations per minute, and his blood pressure normalized.

Upon chart review it was determined that the patient had visited two local hospitals in the prior two days for similar although less severe decompensation of his known mental illness. He was also noted at those times not to be taking his prescribed aripiprazole. He had been given one dose of aripiprazole in the ED the day before the index presentation, but according to family had not taken any since, and as previously noted had not taken his aripiprazole for 3–4 weeks. He was not known to take any other medications. He had been known to use marijuana and cocaine in the past. In 2018 his tetanus was noted to be up to date, but it was unclear when it would need to be updated.

The patient had a long history of medication noncompliance and trialing of multiple antipsychotic medications. Earliest available records in 2017 showed that he was diagnosed with schizophrenia at that time and was initially started on risperidone. He had immediately stopped taking the risperidone and hydroxyzine after discharge and had a long history of re-admissions for decompensations when not taking his medications.

An initial workup was notable for a normal blood glucose and normal electrolytes with the exception of a potassium of 2.5 millimoles per liter (mmol/L) (reference range: 3.6–5.2 mm/L). Magnesium was 1.8 mg per deciliter (dL) (1.7–2.2 mg/dL). An electrocardiogram revealed a QTc-interval of 607 milliseconds (ms) (440–460 ms). This became more pertinent as the patient required more sedation as the effect of the ketamine wore off and he became restless and yelled at staff. He no longer mumbled or spoke in gibberish and had begun clearly pronouncing words in English and in a foreign language as he became more aggressive with staff. No further extension posturing was noted.

The patient was given several more doses of midazolam for a total of 12.5 mg in the ED, followed by a total of 8 mg of lorazepam for longer acting sedation. He also required physical restraints while in the ED. Potassium replacement along with 2 grams of magnesium was initiated, and antipsychotics were avoided due to his prolonged QTc-interval. Just before admission and transfer out of the ED, his repeat electrocardiogram showed a QTc-interval of 411 ms. He remained afebrile and had no leukocytosis. After transfer up to the floor, he required additional midazolam. The patient was admitted initially to the medicine service, given his hypokalemia, but was soon transferred to the mental health unit upon its resolution.

**CPC-EM Capsule**

What do we already know about this clinical entity?

*Opisthotonos, an unusual clinical condition characterized by extreme rigidity and curvature of the back, is not a known side effect of ketamine.*

What makes this presentation of disease reportable?

*There are no reported cases of the opisthotonos associated with ketamine in adults and only a few are reported in infants.*

What is the major learning point?

*With increasing use of this agent providers should be aware of this possible side effect so that they may readily treat it. Midazolam appeared to be effective in this case.*

How might this improve emergency medicine practice?

*As with most medications in our armamentarium increasing awareness of adverse effects will potentially improve the quality of our care.*

## DISCUSSION

Dystonic reactions are relatively rare after ketamine administration. Our patient experienced opisthotonos within minutes of receiving ketamine intramuscularly making ketamine administration the most likely etiology. Opisthotonos, a severe dystonic reaction involving hyperextension and spasticity of the neck and back, has been reported rarely in infants receiving ketamine as anesthesia but is otherwise not seen as a reaction to ketamine administration in the medical literature.^2^ It has been reported in the veterinary literature in multiple other species.^3^ Side effects of ketamine that most providers are aware of include emergence reactions, vomiting and, rarely, laryngospasm.

Opisthotonos is often associated with tetanus but has also been seen in other illnesses such as kernicterus, neurosyphilis, and meningoencephalitis, with administration of phenothiazines and propofol, and poisoning involving strychnine.^4^–^8^ The patient’s presentation and rapid recovery suggest against any of these alternative etiologies. Opisthotonos has been reported in intoxication with the other major arylcyclohexamine N-methyl-D-aspartate (NMDA) receptor antagonists (other than ketamine) such as phencyclidine and methoxetamine, making it biologically plausible that ketamine could have caused the reaction.^9^

The NMDA receptor antagonists are also known to decrease dopamine reuptake and increase dopamine release, as well as inhibit acetylcholine receptors and decrease its release.^10^,^11^ These effects might be expected to decrease dystonic reactions, as medications typically associated with dystonic reactions are dopamine receptor antagonists and these reactions can be treated with anticholinergics. However, there is some suggestion that dopamine receptor antagonists induce a super-sensitivity in the post-synaptic receptor and that this may account for part of the mechanism for dystonic reaction.^12^ Our patient was started on a dopamine antagonist during hospitalization and did not have return of opisthotonos or other dystonic reaction. Lastly, psychostimulants such as amphetamines and related compounds, known to induce movement disorders, are also occasionally associated with dystonic reactions despite increasing dopamine release.^13^ Our patient had a drug screen done as an inpatient that was positive only for benzodiazepines, which we had given him.

Suffice it to say that the mechanisms of dystonic reactions are complex and incompletely elucidated. Regardless, the proximity of the reaction to the administration of ketamine and the absence of other more likely causes make ketamine the most likely trigger in our patient. He was effectively treated with midazolam. The benzodiazepines might be expected to be effective as they are for dystonias in general (as well as for emergence reactions). Diphenhydramine and benztropine would also be options for treatment.^14^

## CONCLUSION

Given the increasing use of ketamine as an agent for controlling agitation more adverse reactions such as the one experienced by our patient could be expected to occur. Characterizing, reporting, and attempting to explain them may enhance our care of patients requiring these treatments.

## References

[1] Scaggs T, Glass D, Hutchcraft M (2016). Prehospital ketamine is a safe and effective treatment for excited delirium in a community hospital based EMS system. Prehosp Disaster Med.

[2] Esposito S, Miller A, Rajon R (2020). Complications of otitis media leads to opisthotonos in a toddler. Am J Emerg Med.

[3] Pachaly J, Werner P (1998). Restraint of the paca (Agouti paca) with ketamine hydrochloride, acetylpromazine maleate, atropine sulfate. J Zoo Wildlife Med.

[4] Das S, von Landeghan F (2019). Clinicopathological spectrum of bilirubin encephalopathy/kernicterus. Diagnostics.

[5] Manjunatha N, Mehta U (2009). Recurrent opisthotonos in catatonia: an atypical presentation. Indian J Med Sci.

[6] Schime I, Tallant E (1959). Tetanus-like reactions to proclorperazine. Report of eight cases exhibiting extrapyramidal disturbances after small doses. JAMA.

[7] Walder B, Tramler M, Margritte S (2002). Seizure-like phenomena and propofol. Neurology.

[8] Ferguson M, Vance M (200AD). Payment deferred: strychnine poisoning in Nicaragua 65 years ago. J Toxicol Clin Toxicol.

[9] Liden C, Lovejoy F, Costello C (1975). Phencyclidine: nine cases of poisoning. JAMA.

[10] Kokkinou M, Ashok A, Howes O (2018). The effects of ketamine on dopaminergic function: meta analysis and review of the implications for neuropsychiatric disorders. Mol Psychiatr.

[11] Sleigh J, Denny B (2014). Ketamine: more mechanisms of action than just NMDA blockade. Anaesth and Crit Care.

[12] Cornett E, Novitch M, Kaye A (2017). Medication-induced tardive dyskinesia: a review and update. Ochsner J.

[13] Asser A, Taba P (2015). Psychostimulants and movement disorders. Front Neuro.

[14] Lewis K, O’Day C (2021). Dystonic reactions.

